# KAGN:knowledge-powered attention and graph convolutional networks for social media rumor detection

**DOI:** 10.1186/s40537-023-00725-4

**Published:** 2023-04-14

**Authors:** Wei Cui, Mingsheng Shang

**Affiliations:** 1 College of Computer Science and Technology, Chongqing University of Posts and Telecommunications, Chongqing, China; 2 Chongqing Key Laboratory of Big Data and Intelligent Computing, Chongqing Institute of Green and Intelligent Technology, Chinese Academy of Sciences, Chongqing, China; 3 School of Electronic Information and Communication Engineering, Chongqing Aerospace Polytechnic, Chongqing, China

**Keywords:** Knowledge graphs, Attention, Graph convolutional networks, Social media, Rumor detection

## Abstract

Rumor posts have received substantial attention with the rapid development of online and social media platforms. The automatic detection of rumor from posts has emerged as a major concern for the general public, the government, and social media platforms. Most existing methods focus on the linguistic and semantic aspects of posts content, while ignoring knowledge entities and concepts hidden within the article which facilitate rumor detection. To address these limitations, in this paper, we propose a novel end-to-end attention and graph-based neural network model (KAGN), which incorporates external knowledge from the knowledge graphs to detect rumor. Specifically, given the post's sparse and ambiguous semantics, we identify entity mentions in the post’s content and link them to entities and concepts in the knowledge graphs, which serve as complementary semantic information for the post text. To effectively inject external knowledge into textual representations, we develop a knowledge-aware attention mechanism to fuse local knowledge. Additionally, we construct a graph consisting of posts texts, entities, and concepts, which is fed to graph convolutional networks to explore long-range knowledge through graph structure. Our proposed model can therefore detect rumor by combining semantic-level and knowledge-level representations of posts. Extensive experiments on four publicly available real-world datasets show that KAGN outperforms or is comparable to other state-of-the-art methods, and also validate the effectiveness of knowledge.

## Introduction

Social media websites have also fostered a variety of rumor, many of which contain misrepresented or even forged content in order to mislead readers and spread quickly. For example, over the last 2 years, social media networks in various countries have been inundated with various rumor about COVID-19. Therefore, in order to maintain social harmony it is highly crucial to detect rumor on these platforms and also regulate them to ensure that the users receive genuine information. The traditional automatic rumor detection methods were based on various hand-crafted linguistic (feature engineering) and semantic features for differentiating between posts documents [1, 2]. With the advent of big data and deep learning, we have seen a shift toward deep-level features. Various deep neural models such as CNN [3], Bi-LSTM [4] and the graph based method [5]are proposed and greatly improve the detection performances.

Even though existing deep neural networks approaches have been successfully used to capture high-level syntax and semantic feature representations of posts content, these approaches do not take into account the external knowledge that is commonly used to judge the authenticity of posts. Generally, posts contents contain many mentions of entities which condense information. A named entity is an individual such as a person, organization, location, or event. A mention is a piece of text that refers to an entity. A named entity could possibly denote different entity mentions because a named entity may have multiple textual forms, such as its aliases, abbreviations and alternate spellings. [6]

**Fig. 1:**
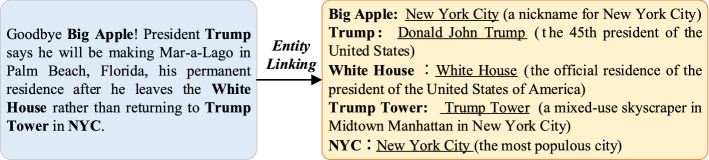
An illustration for entity linking. Entity mentions detected from text are in boldface; By entity linking and disambiguation, the entity mentions are mapped to correct entities which are underlined

As seen in Fig. 1, a post contains the following ambiguous entity mentions: “Big Apple”, “Trump”, “White House”, “Trump Tower”, and “NYC”. When reading the text, one realizes that “Trump” is a person, “Big Apple”, “White House”, “Trump Tower” and “NYC” are geographical locations, and that “Trump” and “Donald Trump" refer to the same person, “Trump” and “Trump” are references to the entity “Donald Trump”. The terms “Big Apple” and “NYC” refer to the same entity “New York City”. These knowledge-level-based judgments and connections help determine the believability of posts. However, the entities and concepts linked with mentions cannot be recognized and comprehended immediately from the content of the posts. As a result, the incorporation of external knowledge is critical for detecting rumor. A knowledge graph is a multi-relational graph, consisting of nodes representing entities and edges representing relationships of various types. On the one hand, the introduction of the knowledge graphs can ensure that each mention in the posts corresponds to the appropriate entity in the knowledge graphs, eliminating the noise caused by ambiguous entity mentions. In addition, knowledge graphs can provide connection information between entities and concepts, which facilitates learning knowledge that is not explicitly stated in the posts text but relevant for rumor detection.

Compared to paragraphs or documents, posts made by users on social platforms do not have sufficient contextual information and suffer from limited word count and incomplete semantics, which leads to semantic ambiguity in posts and poses a significant challenge for short text classification. To resolve this issue, we extract the set of entities and the set of entity-related concepts from the knowledge base(KBs) to enrich the semantics of the text, but some improper entities and concepts are easily introduced due to the ambiguity of entities or the noise in KBs. We therefore propose to use an attention mechanism to inject knowledge into the text in a hierarchical manner, i.e. injecting conceptual knowledge into entities first, and then entities into the text, as a way to filter useful knowledge.

Most of the current work does not consider the implicit connections between knowledge, which may be useful for classification. Therefore, we consider the use of graph structures to establish long-range semantic relations between knowledge, ie. Knowledge share within a sentence on the one hand, and between different posts in the corpus on the other.

Specifically, we propose a Knowledge-Powered Attention and Graph Neural Networks (KAGN) for rumor detection by combining the textual information and knowledge concepts into a unified deep model. To fully utilize external knowledge, we first identify entity mentions in the post contents and then obtain corresponding entities via external knowledge graphs such as Wikidata [7], Probase [8], Freebase [9], and YAGO [10]. Then, as supplementary information, we extract the concepts of each entity. (2)To facilitate the fusion of knowledge, we perform feature extraction from both local and global perspectives. From the local perspective, we calculated the weight distribution of each concept to the same entity using the attention mechanism to consider the granularity of concepts and the relative importance of concepts. Furthermore, taking into account the different contribution of each entities to the posts text, we designed the attention mechanism to determine the semantic similarity between the text and entities. Taking a global view, we built a heterogeneous graph with nodes representing posts, entities, and concepts, and used graph convolutional neural networks to focus on long-range interconnectedness knowledge. (3)Finally, post text representations incorporating entity and knowledge concepts are fed into fully connected layers to predict the authenticity of posts.

The major contributions of this paper are summarized as follows:

- We propose a novel end-to-end unified deep model called KAGN incorporating entities and concepts information derived from knowledge graphs for detecting rumor.
- KAGN utilizes attention mechanisms to hierarchically and effectively inject external entity and conceptual knowledge into the text, and employs graph convolutional networks to mine long-range semantic connections within and between texts, jointly modeling text and knowledge information from both local and global perspectives.
- We conduct extensive experiments on four standard datasets for rumor detection. The results show that KAGN outperforms or is comparable to the state-of-art methods, and the ablation study has demonstrated that KAGN is effective in rumor detection analysis.

### Related works

In this section, we briefly review the work related to the proposed model. We mainly focus on the following topics: rumor detection, knowledge graphs, attention mechanism, graph neural network.

### Rumor detection

#### Social-based rumor detection

Social environment for posts contains an abundance of information, such as the interaction patterns of the users, the dissemination patterns, and the credibility of the posts. Ma et al. [11] propose a kernel-based method to capture high-order patterns of microblog posts diffusion with propagation trees, which provide valuable clues on how a post is diffused and developed over time.Liu et al. [12]modeled the propagation path as multivariate time series, and applied both recurrent and convolutional networks to capture the variations of user characteristics along the propagation path. Wu et al. [13] proposed a random walk graph kernel to model the propagation trees of messages to improve rumor detection. Sitaula et al. [14]analyzed the history of association between authors and rumor, as well as the number of authors of posts to detect rumor on the internet.

#### Content-based rumor detection

A large number of researchers have looked for important clues to distinguish rumor from credible posts through semantic, style and knowledge graphs of posts content. Various deep models, such as the architecture of LSTM [15], graph convolutional network [16], gated GNN [17], generative adversarial network (GAN) [18], deep convolutional neural network [19], event adversarial network [20], and hybrid convolutional neural network [21] are used to extract potential textual and visual features of posts content. Approaches based on knowledge graphs have also been investigated for rumor detection. [22] propose a Knowledge-driven Multimodal Graph Convolutional Network (KMGCN) to jointly model the semantic representations of textual information, knowledge concepts and visual information for fake news detection. The authors of [23] introduced a KGs(Knowledge Base) for factchecking claims by collecting data from popular fact-checking websites and exploring additional information from DBpedia. Furthermore, researchers have proposed interpretable methods for detecting rumor using KGs [24].

### Knowledge graphs

Google officially released the Knowledge Graphs in 2012 [25]. A knowledge graph is a large-scale semantic network that generates new knowledge by acquiring information and integrating it into a knowledge base and then reasoning about it, which contains a large amount of entities, attributes, and semantic information between entities. Knowledge graphs have been widely used in risk control anti-fraud, credit auditing, accurate advertising delivery, search engines, personalized recommendation systems and question and answer systems [26–28]. Knowledge graphs generally use triples to record and store entity relationships, and the hidden attributes of entities and their relationships with other entities can be mined through knowledge graphs embedding learning, and the knowledge graphs triples are represented as low-dimensional vectors [29].

A named entity is an individual, such as a person's name, a place name, or an organization’s name. An entity mention is a name string that appears in the text to refer to the entity. To extract named entities from text, two main tasks are involved: named entity recognition tries to find every fragment of text that mentions a named entity. Named entity linking is divided into candidate entity generation, which is based on retrieving the knowledge base to get all the eponymous entities to form a candidate entity set, and candidate entity disambiguation, which is a method to find the target entity from the candidate entity set that matches the current context.

### Attention mechanism

Bahdanau et al. [30] first used an attention mechanism in a machine translation task, which was mainly based on the Encoder-Decoder framework, where the attention mechanism weighted the source sentence features to focus on those that were important for the current translation and ignored those that were not. Yang et al. [31] proposed a hierarchical attention mechanism, which introduced an attention mechanism at the word level to get important sentence features and introducing an attention mechanism to get important document features at the sentence level to achieve document classification. The Transformer model proposed by Google Vaswani et al. [32] is an automatic translation model, which proposes a self-attention mechanism approach, which is one of the representative approaches in the development of attention mechanism. Wu et al. [33] combine word embeddings with contextual embeddings of words captured using a self-attentive mechanism, and then capture semantic features by convolutional neural networks for text classification. Liu et al. [34] proposed to use an attention mechanism to assign different weights to the information output from the hidden layer of the bidirectional LSTM to obtain local features and global semantics of phrases to improve the classification accuracy. Ma et al. [35] proposed a Global–Local Mutual Attention (GLMA) model for the text classification problem, which introduces a mutual attention mechanism for mutual learning between local semantic features and global long-term dependencies. Guo et al. [36] proposed a multi-scale self-attentive mechanism model where the selfattentive mechanism is introduced into the multi-scale structure to extract different scale features of the text. In addition, the multi-head self-attention mechanism in Transformer idea is also combined with multi-scale to let each head extract different scale information of the text.

### Graph neural networks

Yao et al. [37] were the first to apply graph convolution to text classification tasks, and proposed the TextGCN model to construct a corpus-level graph for the entire dataset using words and text as nodes, and to learn both word representation and text representation using standard graph convolutional networks. Liu et al. [38] proposed a tensor graph neural network model for coordinating and integrating multi-graph heterogeneous information, constructing a text graph tensor to describe semantic, syntactic, and sequential contextual information, and then performing intra-graph and inter-graph propagation on the text graph tensor. Hu et al. [39] introduced a two-layer attention structure in a heterogeneous graph neural network to obtain key in-formation at different granularity levels and reduce the influence of noisy information. Zhang et al. [40] proposed TextING to construct a text-level edge weight matrix and use Gated Graph Neural Network (GGNN) to update the word node representation in the message passing phase. Giannis et al.[41] proposed MPAD, which introduces a text node in the construction of the text-level graph and establishes a connection with all words to obtain global statistics, and puts the word representation through a self-attention mechanism to obtain a temporary text representation in the read-out phase, and performs a join operation with the updated representation of the text node in the graph as the final text representation for classification.

### The proposed method

In this section, we mainly introduce the proposed Knowledge-Powered Attention and Graph Neural Networks (KAGN) in detail. We first describe the problem definition, and then, we introduce the overall framework of KAGN. The details of the proposed model are shown in the following sections. The symbols appeared in this paper and their meaning is interpreted in Table 1.

**Table 1:** Summary of the main notations

Notation	Explanation
$D$	The training news samples
$P$	A piece of news composed of a sequence of words
$EP$	The relevant entities of P
$CE$	The entity-related concepts of EP
$p$	The representation of P
$EP^{\prime}(q^{\prime})$	The representation of entities
$CE^{\prime}\left( {r^{\prime}} \right)$	The representation of entity-related concepts
$e_{i}$	An entity in knowledge base
$ce_{i}$	Set of all concepts for an entity
$e_{i}^{\prime }$	Embedded representation of an entity
$ce_{i}^{\prime }$	Embedded representation of a concepts set
$c_{j}$	A concept of $ce\left( {e_{i} } \right)$
$\tilde{q}$	Entity representation incorporating conceptual knowledge
$\tilde{p}$	Textual representation of news incorporating entity and concept knowledge
$\hat{p}$	Textual representation of news obtained by gating mechanism
$G$	The post-entity-concept graph
$EN$	The unique entities nodes sets of graph G
$en_{i}$	A uniquely numbered entity node
$CN$	The unique concepts nodes sets of graph G
$cn_{i}$	A uniquely numbered concept node

### Task definition

A rumor detection task can be defined as a binary classification problem, which aims to classify a post in social media as rumor or not. The goal of our model is to identify whether a post is fake or not at the post-level. Let $P = \left\{ {w_{1} , \ldots ,w_{n} } \right\}$ is a post which consists of a sequence of words. For every post $P$, where one or several words may be associated with an entity $e_{i}$ in the knowledge graphs. In addition, each entity $e_{i}$ are linked to many concepts in the knowledge graphs. The concepts of the entity $e_{i}$ is defined as “entity context” ce $\left( {e_{i} } \right)$. Formally, given a rumor post $P = \left\{ {w_{{\text{i}}} } \right\}$ as well as the relevant entities $EP = \left\{ {e_{i} } \right\}$ and entity concepts $CE = \left\{ {ce\left( {e_{i} } \right)} \right\}$. We need to learn a model $f\left( {y|P,EP,CE;\Theta } \right)$ where $y$ is class label and $\Theta$ represents all parameters of the model.

### Overall framework

**Fig. 2:**
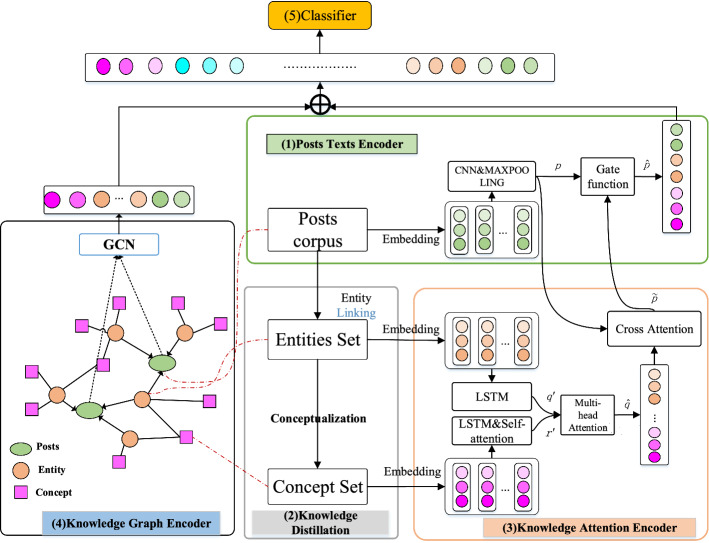
The overall framework of KAGN. (1)Post texts encoder uses Word2Vec and CNN to get the posts texts representation. (2)Knowledge distillation module extract entities and concepts sets of posts contents from knowledge graphs. (3)Knowledge attention encoder employs Bi-LSTM, self-attention and multi-head attention to obtain the representations of entities and concepts, which are then fused with posts texts representation using a gate control mechanism (4)Knowledge graphs encoder creates a graph of posts texts, entities, concepts to learn the global knowledge using GCN. (5)The results of (1), (3) and (4) are concatenated and passed to a fully connected softmax layer whose output is the probability distribution over all the categories

Our model KAGN is a knowledge-enhanced deep neural network to model the semantic-level representations in a unified framework. We provide a brief overview of our model before detailing it. Figure 2 shows the framework of KAGN, which mainly consists of the following components:

Posts texts encoder. The module encodes the short rumor posts texts by Word2Vec and CNN to produce text representation.

Knowledge distillation module. The module retrieves conceptual information relevant to the posts from KGs. The background knowledge distilled from a real word knowledge graphs can complement the semantics representation of short texts of rumor posts. Furthermore, the conceptual information extracted from entities can provide additional evidence to enhance rumor detection.

Knowledge encoder. The knowledge encoder is made up of two parts: knowledge attention encoder and knowledge graphs encoder. To discover local and longdistance knowledge semantics information, two methods are used. To begin, the knowledge attention encoder employs self and cross attention mechanisms to obtain the joint representations of entities and concepts. Following that, knowledge graphs encoder models the posts' texts, entities, and concepts as directed graphs based on the knowledge graphs. A graph convolutional network is used to obtain the local and global semantic-level features for each post based on the constructed graph. To aggregate the nodes of the graph and obtain the representation vector of each post, we use two GCN layers and a global mean pooling. Finally, we fuse the knowledge information by concatenating the outputs of the two approaches.

Classification module. This component combines the representations of the texts encoder and the knowledge encoder to perform the final downstream rumor classification learning. We use an output layer to acquire the probability of each class label.

### Posts texts encoder

The posts texts encoder aims to generate the text representation of posts. To model sentences, RNN [42], CNN [43], and hybrid models have been widely used. To learn the semantics of posts, we use CNN-based models as the basic component of the model in this work.

Given a piece of post $P = \left\{ {w_{1} ,w_{2} , \cdots ,w_{L} } \right\}$ of length $L$, each word $w_{i}$ is projected into a continuous word embedding $w_{i}^{\prime }$ from a word embedding matrix $M \in {\mathbb{R}}^{v \times d}$ where $v$ is the vocabulary size and $d$ is the embedding dimension. Then, we obtain the post vectors $P^{\prime} = \{ w_{1}^{\prime } ,w_{2}^{\prime } , \cdots ,w_{L}^{\prime } \} \in {\mathbb{R}}^{d \times L}$, where $w_{i}^{\prime } \in {\mathbb{R}}^{d \times 1}$ is the embedding of the $i$-th word in the post. A convolutional kernel $k \in {\mathbb{R}}^{d \times h}$ is applied on the word embedding matrix $P^{\prime}$ to obtain a feature map. Specifically, a feature $e_{i}$ generated from a sub-matrix $w^{\prime}_{i:i + h - 1}$ by

1 $$e_{i} = f\left( {k * w^{\prime}_{i:i + h - 1} } \right)$$

where $h\left( {h \le n} \right)$ is the receptive filed size of convolutional kernel, $f\left( \cdot \right)$ is non-linear transformation function, ${*}$ is the convolution operator. After applying the convolutional filter to every possible position in $P$, a feature map is obtained,

2 $$e = \left[ {e_{1} ,e_{2} , \ldots ,e_{L - h + 1} } \right] \in {\mathbb{R}}^{{\left( {d/3} \right) \times \left( {L - h + 1} \right)}}$$

Next, we apply a max-over-time pooling operation over the feature map $e$ to obtain

3 $$\hat{e} = {\text{max}}\left( e \right) \in {\mathbb{R}}^{d/3}$$

In this manner, one feature is extracted from one filter. Convolutional kernel with varying receptive filed can extract sentence features from different angles, so the CNN layer uses three d/3(out channel of Conv1d) kernels with different kernel size (3, 4 and 5) respectively. Finally, we concatenate all kinds of filters' outputs to form $p \in R^{d}$ as the final representation of the post $P$.

### Knowledge distillation

Background knowledge derived from a real-word knowledge graphs can be used to supplement the semantic representation of short post texts. Furthermore, the conceptual information extracted from entities can be used to provide additional evidence to aid in the detection of rumor. This module's specific goal is to retrieve relevant knowledge from knowledge graphs.

**Fig. 3:**
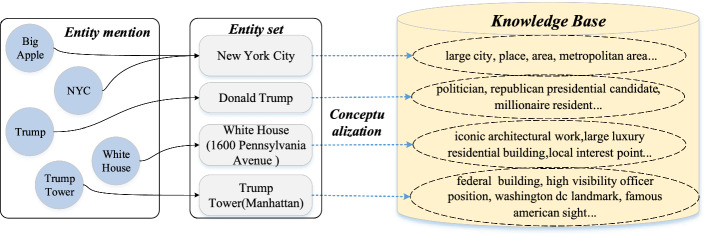
An illustration for entity conceptualization. Conceptual knowledge extracted from a knowledge base for each entity are shown via a dashed arrow line and oval

We hope to find a concept set $$CE$$ relevant to a given post text. The knowledge distillation process consists of two steps in Fig. 3. Given the short text content of posts, many entity linking methods, such as EDEL [44], Rel-Norm [45], can be used to connect ambiguous entity mentions in a text to the correct entities $$e$$ in the knowledge graphs. Then, for each identified entity $$e \in EP$$. We obtain its conceptual information by conceptualization from an existing knowledge graphs, such as Wikidata [7], Probase [8]. For example, as shown in Fig. 3, by entity linking and disambiguation, we obtain the entity set $$EP = \{$$ New York City, Donald Trump, White House(1600 Pennsylvania Avenue), Trump Tower(Manhattan) $$\}$$. The entities in the $$EP$$ are then conceptualized in order to construct concept sets, such as ce(New York City) = (large city, place, metropolitan area), ce(Donald Trump$$) = ($$ politician, republican presidential candidate, millionaire resident), which are acquired from external knowledge graphs. Given a piece of post $$P$$, we can distill knowledge from the knowledge graphs and obtain a set of concepts $$CE = \left\{ {ce\left( {e_{1} } \right),ce\left( {e_{2} } \right), \ldots ,c\left( {e_{n} } \right)} \right\}$$ for all entities.

## Knowledge attention encoder

### Knowledge encoder

Prior knowledge obtained from external knowledge base provides richer information and reduces ambiguity caused by entity mentions in posts. Given a piece of post, entities and entity-related concepts in the post can help to improve performance of rumor detection. The extracted entities sequence $EP$ and entity concepts sequence $CE$ are embedded by Word2Vec [46], given a post $P$, we obtain the entities embedding and concepts embedding

4 $$q^{\prime} = EP^{\prime} = \left\{ {e_{1}^{\prime } ,e_{2}^{\prime } , \cdots ,e_{n}^{\prime } } \right\},EP^{\prime} \in R^{n \times d}$$

5 $$r^{\prime} = CE^{\prime} = \left\{ {ce^{\prime}\left( {e_{1} } \right),ce^{\prime}\left( {e_{2} } \right), \cdots ,ce^{\prime}\left( {e_{n} } \right)} \right\} = \left\{ {ce_{1}^{\prime } ,ce_{2}^{\prime } , \cdots ,ce_{n}^{\prime } } \right\},CE^{\prime} \in R^{n \times m \times d}$$

where $d$ is the embedding dimension. The entity embedding $e_{i}^{\prime }$ and concept embedding $ce_{i}^{\prime }$ are calculated as follows. Note that an entity or a concept can typically involve a phrase consisting of multiple tokens, rather than a single word. Therefore, we employ a sequential bidirectional LSTM model to calculate the phraselevel representation of an entity or a concept from its word embedding. For example, given a concept $c_{j}$ of $ce\left( {e_{i} } \right) = \left\{ {c_{1} , \cdots c_{j} , \cdots c_{{\text{m}}} } \right\}$ (m denotes the index of concept phrase for entity $e_{i}$), the concept representation at the phrase-level is denoted as $c_{j}$, and the word-level is $\left\{ {c_{{\text{j}}}^{\left( 1 \right)} , \cdots ,c_{{\text{j}}}^{{\left( {\text{L}} \right)}} } \right\}$ (L denotes the padded word length of the concept phrase $c_{j}$). We first obtain the word-level embedding $\left\{ {c_{1}^{\prime \left( 1 \right)} , \cdots ,c_{1}^{{\prime \left( {\text{L}} \right)}} ;c_{1}^{\prime \left( 1 \right)} , \cdots ,c_{1}^{{\prime \left( {\text{L}} \right)}} ;c_{1}^{\prime \left( 1 \right)} , \cdots ,c_{1}^{\prime \left( 1 \right)} } \right\}$ of ce $\left( {e_{i} } \right)$ via Word2Vec. Then, for the phrase-level embedding, we use a sequential LSTM model and max-pooling to calculate $ce^{\prime}\left( {e_{i} } \right) = ce_{i}^{\prime } \in R^{m \times d}$ from its word-level representation,

6 $$ce_{i}^{\prime } = {\text{Concat}}\left( {{\text{Maxpool}}\left( {{\text{LSTM}}\left\{ {c_{j}^{\prime \left( 1 \right)} , \cdots ,c_{j}^{{\prime \left( {\text{L}} \right)}} } \right\}} \right)} \right),{\text{j}} = 1 \ldots m$$

similarly, we get $e_{i}^{\prime } \in R^{1 \times d}$.

#### Knowledge-aware attention

To effectively integrate external knowledge after obtaining the embedding of entities $q^{\prime}$ and entity-related concepts $r^{\prime}$, we design multi-head attention and cross-attention networks to distinguish the relative importance of knowledge. An entity has multiple different concepts in a post text, in order to select the proper concepts of an entity according to the context, we propose Concept to Entity(C-E) attention to measure the importance of each concept with respect to the entity. We apply multi-head attention to build the connection between entities and concepts. The formula of multi-head attention is as follows:

7 $$\begin{gathered} {\text{Attention}}\left( {Q,K,V} \right) = {\text{softmax}}\left( {\frac{{QK^{T} }}{{\sqrt {d_{k} } }}} \right)V \hfill \\ {\text{MultiHeadAttention}}\left( {Q,K,V} \right) = {\text{Concat}}\left( {{\text{Attn}}_{1} , \cdots ,{\text{Attn}}_{H} } \right) \hfill \\ \end{gathered}$$

where queries, keys and values are packed together into matrices $Q,K$ and $V,d_{k}$ is the dimension of queries and keys, $H$ is the number of heads. Specifically,

8 $$\begin{gathered} Q = W_{Q} q^{\prime},K = W_{K} r^{\prime},V = W_{V} r^{\prime} \hfill \\ \alpha = {\text{softmax}}\left( {\frac{{QK^{T} }}{{\sqrt {d_{k} } }}} \right),\tilde{q} = \alpha V \hfill \\ \end{gathered}$$

We treat the entities representation $q^{\prime}$ as queries to attend to the concepts representation $r^{\prime}$ in order to compute attention scores, which might capture dependencies between entities and concepts. $\tilde{q}$ denotes a new entity representation that incorporates conceptual knowledge. C-E attention has a similar effect to feature selection. It is a soft feature selection that gives a higher weight to a critical concept and a low weight (near zero) to a trivial concept. To account for the relative importance of entities, we propose the Entity to Post (E-P) attention metric, which measures each entity's contribution to the post text. We define E-P attention for each entity as follows.

9 $$\begin{gathered} \beta = {\text{softmax}}\left( {W_{2}^{T} f\left( {W_{1} {\text{concat}}\left[ {p;\tilde{q}} \right]} \right)} \right) \hfill \\ \tilde{p} = \beta \tilde{q} \hfill \\ \end{gathered}$$

In E-P attention, we apply cross attention to build the connection between post and entity. $f\left( \cdot \right)$ is a non-linear activation function such as hyperbolic tangent transformation and softmax is used to normalize attention weight of each entity. $W_{1} \in {\mathbb{R}}^{2d \times h}$ is a weight matrix and $W_{2} \in {\mathbb{R}}^{h \times 1}$ is a weight vector. $\beta$ denotes the weight score of attention from entity towards the post. A larger $\beta_{i}$ means that the $i$-th entity is more related to the post. The score is applied to aggregate the entities's representation to form a new post representation $\tilde{p}$.

In order to form a joint representation, a learnable gating function is employed to combine original post representation and the new one. Formally,

10 $$\alpha = \sigma \left( {{\text{W}}_{3} {\text{concat}}\left( {p;\tilde{p}} \right)} \right)$$

11 $$\hat{p} = p \odot \alpha + \tilde{p} \odot \left( {1 - \alpha } \right)$$

where $\sigma \left( \cdot \right) = \frac{1}{{1 + {\text{exp}}\left( \cdot \right)}}$ is sigmoid activation function, ${\text{W}}_{3} \in {\mathbb{R}}^{2h \times 1}$ is learnable parameters of the fusion gate, $\odot$ denotes element-wise multiplication. $\alpha$ is a gating vector to trade-off information from the two sources and its elements are in[0,1]. $\hat{p}$ is the final textual representation of the post that incorporates entity and concept knowledge.

### Knowledge graphs encoder

This section presents our proposed knowledge graphs encoding scheme. We first detail how a text-graph is constructed from the posts texts, entities and concepts, and then present the graph convolutional encoder for obtaining knowledge structure in-formation that encode the textual information.

#### Text-graph construction

To better exploit global and long range knowledge semantic relationships in the posts texts, we build a heterogeneous post-entity-concept graph $$G = \left\{ {V,E} \right\}$$, where $$V$$ represents the nodes including posts-texts nodes $$D = \left\{ {P_{1} , \ldots ,P_{{\text{n}}} } \right\}$$, unique entities nodes sets $$EN = \left\{ {en_{1} , \ldots ,en_{z} } \right\}$$ and unique concepts nodes sets $$CN = \left\{ {cn_{1} , \ldots ,cn_{{\text{b}}} } \right\}$$, and $$E$$ denotes the edges. As shown in Fig. 2, the connection between concepts and entities is undirected, allowing for higher-level knowledge sharing and flow, whereas the connection between entities and posts is directed, i.e., from entities to posts, to reduce interference between rumor posts and non-rumor posts. There are two types of typical relations: (1)local relation, e.g.,concept $$\to$$ entity $$\to$$ post $$\leftarrow$$ entity, which was capable of learning local knowledge; and (2)long-range relation, e.g., concept $$\to$$ entity $$\to$$ concept $$\to$$ entity $$\to$$ post, which has the ability to perceive knowledge from a distance. To encode these two types of relations into the node representation, the graph nodes can aggregate both local and global longrange semantic relationships among posts texts, entities and concepts from graph structure. Specifically, for each post $$p \in D$$ we first select $K$ entities with the highest possibility values with the entity link tool as entity nodes, the entities of all posts are de-duplicated to obtained unique entities nodes sets $$EN = \left\{ {en_{1} , \ldots ,en_{z} } \right\}$$, and then we build edges between the $$EN$$ and the post texts set $$D$$. To incorporate global semantics among post texts and concept, we further build edges between entities set and its semantic concepts set. In this work, for every entity, we select the top $M$ concepts with the highest probability score based on the Microsoft Concept Tagging Model [47], the concepts of all entities are de-duplicated to obtained unique concepts nodes sets $$CN = \left\{ {cn_{1} , \ldots ,cn_{b} } \right\}$$. Figure 2 shows the text-entity-concept graph $$G = \left\{ {V,E} \right\}$$ constructed from the text (Fig. 3).

#### Graph convolutional encoder

After constructing the text-entity-concept graph, GCN which is effective in capturing high-order neighborhood information, is applied to learn the representations of posts texts that aggregate high-order semantic information. Note that we employ the distributed Word2Vec representation to obtain the embeddings of posts texts, entities and concepts nodes, which denoted as $D^{\prime} = \left\{ {p_{1}^{\prime } , \ldots ,p_{n}^{\prime } } \right\} \in {\mathbb{R}}^{n \times s \times d} ,EN^{\prime} = \left\{ {en_{1}^{\prime } , \ldots ,en_{z}^{\prime } } \right\} \in {\mathbb{R}}^{z \times s \times d} ,CN^{\prime} = \left\{ {cn_{1}^{\prime } , \ldots ,cn_{b}^{\prime } } \right\} \in {\mathbb{R}}^{b \times s \times d}$, and then initialize them by bidirectional ${\text{LSTM}}\left( {{\text{BiLSTM}}} \right)$ [48] network to get semantic vector representation $X$ as initial nodes feature matrix of graph. In this way, the input embeddings of the nodes are in the same semantic space, thus we can directly apply GCN on the graph.

12 $$\begin{gathered} \user2{\tilde{D}^{\prime}} = {\text{BiLSTM}}\left\{ {p_{1}^{\prime } , \ldots ,p_{n}^{\prime } } \right\} \in {\mathbb{R}}^{n \times s \times 2d} \hfill \\ \widetilde{{\user2{EN^{\prime}}}} = {\text{BiLSTM}}\left\{ {en_{1}^{\prime } , \ldots ,en_{z}^{\prime } } \right\} \in {\mathbb{R}}^{z \times s \times 2d} \hfill \\ \widetilde{{{\varvec{CN}}}}^{\prime } = {\text{BiLSTM}}\left\{ {cn_{1}^{\prime } , \ldots ,cn_{b}^{\prime } } \right\} \in {\mathbb{R}}^{b \times s \times 2d} \hfill \\ X = {\text{ Maxpool}}\left\{ {{\text{Concat}}\left( {\user2{\tilde{D}^{\prime}};\widetilde{{{\varvec{EN}}}}^{^{\prime}} ;\widetilde{{{\varvec{CN}}}}^{^{\prime}} } \right)} \right\} \hfill \\ \end{gathered}$$

where $n,z$ and $b$ represent the number of posts, entities and concepts respectively, $s$ denotes the padded word length of posts, entities and concepts, $d$ is the embedding dimension.

Formally, we consider the text-entity-concept $G = \left\{ {V,E} \right\}$,where $V$ and $E$ represent the set of nodes (including post texts, entities and concepts) and edges respectively. We introduce an adjacency matrix $A$ of $G$ and its degree matrix $D$, where $D_{ii} = \sum_{j} A_{ij}$ the diagonal elements of $A$ are set to 1 with self-loops. Each node is associated with a d-dimensional feature vector and we use a feature matrix $X \in R^{{\left( {n + z + b} \right) \times 2d}}$ to represent the initial features of all vertices, where the $i_{th}$ row corresponds to the feature vector of the $i_{th}$ node. Based on the adjacency matrix $A$ and the degree matrix $D$, each GCN layer input feature matrix $X^{\left( l \right)} \in R^{{\left( {n + z + b} \right) \times 2d^{\left( l \right)} }}$ (the input feature matrix of first layer is $X^{\left( 0 \right)} \in R^{{\left( {n + z + b} \right) \times 2d^{\left( 0 \right)} }}$) and output a higher order feature matrix $X^{{\left( {l + 1} \right)}} \in R^{{\left( {n + z + b} \right) \times 2d^{{\left( {l + 1} \right)}} }}$ for vertices as follows:

13 $$Z^{{\left( {l + 1} \right)}} = \sigma \left( {D^{{ - \frac{1}{2}}} \left( {I + A} \right)D^{{ - \frac{1}{2}}} X^{\left( l \right)} W} \right)$$

where $W \in R^{{2d^{\left( l \right)} \times 2d^{\left( l \right)} }}$ is a weight matrix that can be learned during training, $I$ is the identify matrix and $\sigma$ is a non-linear activation function, e.g. a ${\text{ReLU}}\sigma \left( x \right) = {\text{max}}\left( {0,x} \right)$ After going through a 2-layer GCN, we get the embeddings $g$ with respect to the post nodes which aggregate semantics from their neighbors in the graph.

### End-to-end model training

After the above procedures, the post text encoder' output $p$, knowledge attention encoder's output $\hat{p}$ and knowledge graphs encoder's output $g$ are concatenated as final features ${\varvec{z}}$ for classification. Then, ${\varvec{z}}$ is fed into a fully connected layer followed by a softmax function to project the final representation into the target space of classes probability:

14 $$P = {\text{softmax}}\left( {{\varvec{W}}_{o} {\varvec{z}} + {\varvec{b}}_{o} } \right)$$

It is trained to minimize the cross entropy loss function:

15 $$J = - \sum\limits_{i \in D} {c_{i} } {\text{log}}\mathcal{P}_{i} + \frac{\lambda }{2}\parallel \Theta \parallel_{2}^{2}$$

where $D$ denotes the overall training corpus, $c_{i}$ refers to the ground truth label for posts $i,\mathcal{P}_{i}$ denotes the probability of the predicted label, $\Theta$ denote the parameters of KAGN, and $\lambda$ is the coefficient of L2 regularizer.

## Experimental

### Datasets

We evaluate the proposed model on four real-world data collections: Twitter15, Twitter16, PHEME and Politifact [10, 49–51] which were originally collected from the most popular social media website. Each sample in Twitter 15 and Twitter 16 datasets is annotated with one of four more finer-grained classes, i.e., non-rumor, false rumor, true rumor, and unverified rumor. Note that the label "true rumor" denotes a post that tells people that a certain post is fake. Each sample in the PHEME and Politifact datasets is labeled as one of two categories, i.e., rumor or non-rumor. For each data set, a graph is constructed from source tweets, entities, and related concepts. The details of these four datasets are reported in Table 2." #" denotes "the number of". The four datasets are available to the public online^1^,^2^.^3^

**Table 2:** Statistics of the datasets

Statistic	Twitter15	Twitter16	PHEME	PolitiFact
# Source tweets	1490	818	2742	815
# Non-rumor	374	205	–	–
# False rumor	370	205	1886	443
# Unverified rumor	374	203	–	–
# True rumor	372	205	856	372
# Users	276663	173487	–	–
# Posts	331612	204820	–	–

### Implementation details

In the process of knowledge extraction, we utilize entity linking tools TagMe [52] to disambiguate entity mentions in posts contents and link them to corresponding entities in the knowledge graphs Wikidata [7]. In the procedure of entity concepts extraction, we retrieve the entities in Microsoft Concept Graph [8] only consider the $isA$ relation. For all models, adam optimizer [53] is adopted for learning, with a learning rate of $0.003$ gradually decreased during the process of training, and the dropout rate is set to 0.5. The batch size is set to 16. The training epochs are set to 50. The word embedding are initialized with the 300 dimensional word vectors, which are trained on domain specific review corpora by Skip-gram algorithm [46]. If a word is unknown, we will randomly initialize its embedding. We also use 300 dimension entity embedding and concept embedding which is initialized by 300 dimensional word vectors. All word embedding, entity embedding and concept embedding are trainable and fine-tuned in the training stage, since we hope to learn task-oriented representation. We use $1{\text{DCNN}}$ with filters of width $\left[ {2,3,4} \right]$ of size 300 for a total of 100.The number of attention heads $h$ is set to 8. The GCN encoder is set to 2 layers. The evaluation metric is accuracy, precision, recall and F1 score which is widely used in text classification tasks [54]. We conduct fivefold cross-validation and hold out $10{\text{\% }}$ instances as the validation data set to tune the hyper parameters, and the remaining datasets is split into training and testing sets in the ratio of 3 to 1.

### Baselines

The experiments on the two datasets use the baselines listed as follows:

- DTC [55]: A decision tree classifier using various handcrafted features extracted by feature engineering to detect rumor.
- RFC [56]: A random forest classifier that selects temporal, structural, and linguistic characteristics.
- SVM-TS [57]: A linear SVM classifier that utilizes the variation of social context features during the rumor propagation over time.
- PTK [10]: A tree-based kernel approach using SVM classifier to evaluate the similarities between propagation tree structures of rumor and non rumor.
- GRU [48]: A RNN-based model was utilized to learn variation features of contextual information about relevant rumor over time.
- BU-RvNN and TD-RvNN: [58]: A RvNN models based on bottom-up and top-down tree structures to capture propagation structural and textural semantics.
- PPC [12]: A recursive and convolutional classifier to model the local and global variation of user features along the propagation path.
- Bi-GCN [59]:A bi-directional GCN model to explore propagation and dispersion characteristics of rumor from both top-down and bottom-up propagation path.
- CNN [60]: CNN learns rumor representations using a convolution network by structuring relevant posts as a fixed-length sequence.
- B-TransE [61]: The B-TransE method incorporates both positive and negative single models to identify fake news based on news content and knowledge graphs.
- KCNN [62]: The KCNN learns semantic-level and knowledge-level representations of news by combining knowledge entities and common sense in news material.

In addition to the above baselines, we design several variants to demonstrate the effectiveness of each component in our model. We will introduce these variants in the results and analysis section.

## Results and analysis

### Comparison of different models

The hyperparameters of all variables in the experiment were determined by validation set. We evaluated these variants by a fivefold cross-validation. Table 3, 4, 5, 6 show the experimental results of our proposed KAGN and all compared baselines approaches on four datasets, respectively. From the Tables, we can draw the following observations:

**Table 3:** Results of comparison with different models on twitter15 datasets

Method	Acc	NR	FR	TR	UR
		F_1	F_1	F_1	F_1
DTC	0.454	0.733	0.355	0.317	0.415
RFC	0.565	0.81	0.422	0.401	0.543
SVM-TS	0.544	0.796	0.472	0.404	0.483
PTK	0.75	0.804	0.698	0.765	0.733
GRU	0.646	0.792	0.574	0.608	0.592
BU-RvNN	0.708	0.695	0.728	0.759	0.653
TD-RvNN	0.723	0.682	0.758	0.821	0.654
PPC	0.842	0.811	0.875	0.818	0.79
Bi-GCN	0.886	0.891	0.86	0.93	0.864
KAGN	0.892	0.868	0.883	0.894	0.927

*NR* non-rumor, *FR* false rumor, *TR* true rumor, *UR* unverified rumor

**Table 4:** Results of comparison with different models on twitter16 datasets

Method	Acc	NR	FR	TR	UR
		F_1	F_1	F_1	F_1
DTC	0.465	0.643	0.393	0.419	0.403
RFC	0.585	0.752	0.415	0.547	0.563
SVM-TS	0.574	0.755	0.42	0.571	0.526
PTK	0.732	0.74	0.709	0.836	0.686
GRU	0.633	0.772	0.489	0.686	0.593
BU-RvNN	0.718	0.723	0.712	0.779	0.659
TD-RvNN	0.737	0.662	0.743	0.835	0.708
PPC	0.863	0.82	0.898	0.843	0.837
Bi-GCN	0.88	0.847	0.869	0.937	0.865
KAGN	0.901	0.864	0.881	0.946	0.908

**Table 5:** Results of comparison with different models on PHEME datasets

Method	Acc	Precision	Recall	F-1
SVM-TS	0.640	0.639	0.621	0.640
DTC	0.691	0.648	0.654	0.650
RFC	0.713	0.660	0.609	0.614
CNN	0.701	0.741	0.707	0.690
GRU	0.737	0.700	0.690	0.692
B-TransE	0.720	0.683	0.606	0.607
KCNN	0.727	0.683	0.642	0.649
KAGN	0.865	0.840	0.829	0.834

**Table 6:** Results of comparison with different models on Politifact datasets

Method	Acc	Precision	Recall	F_1
SVM-TS	0.669	0.746	0.683	0.647
DTC	0.749	0.748	0.745	0.745
RFC	0.741	0.747	0.736	0.736
CNN	0.701	0.741	0.707	0.690
GRU	0.711	0.708	0.705	0.704
B-TransE	0.769	0.774	0.766	0.764
KCNN	0.783	0.785	0.782	0.780
KAGN	0.879	0.877	0.878	0.875

It is evident that the performance of models based on hand-crafted features using traditional machine learning methods (i.e., DTC, RFC, SVM-TS, and PTK) seems to be unsatisfactory, probably because these methods lack generalization due to the difficulty in capturing useful features. While SVM-TS and PTK are better than DTC and RFC on Twitter15 and Twitter16 datasets, because they employ propagation structures or social context features, they remain clearly inferior to those not relying on feature engineering. However, SVM-TS achieves lower results on PHEME and Politifact. This is probably because SVM-TS is limited to deal with long sentences in the datasets.

We observe that deep learning methods (e.g. PPC, RvNN, Bi-GCN, CNN and GRU) outperform those traditional machine learning methods that employ handcrafted features on four datasets which suggests the superiority of feature extraction of deep neural networks. It appears that deep learning methods are capable of learning the underlying deep features of rumor. On Twitter15 and Twitter16, Bi-GCN achieves optimal performance using the structure learning capability of graph convolutional networks due to the consideration of the deep propagation and wide scattering features of rumor. Furthermore, methods that use both news content and external knowledge consistently outperform methods that merely utilize news content on PHEME and Politifact, such as KCNN > B-TransE > GRU, CNN, RFC, DTC, and SVM. This shows that models can successfully incorporate external knowledge and improve detection performance significantly.

Moreover, as shown in Tables 3, 4, 5, 6, our proposed KAGN method has achieved the best performance compared with all other baselines on four datasets. Specifically, our proposed model achieves performance improvement by 0.6% on Twitterr15, 2.1% on Twitterr16, 13.8% on PHEME, and 9.6% on Politifact. This demonstrates that our proposed framework can effectively capture the global semantic relations of the text contents in rumor, which is helpful for rumor detection. Three factors explain the superior performance of KAGN. (1) KAGN uses the concept of knowledge to enrich the semantic information of post text with the help of KGs. (2) Our model is able to focus on more important entity and concept knowledge and effectively fuse them into texts representation due to the attention mechanism. (3) Graph convolutional network can capture the intrinsic dependencies among implicit knowledge to obtain more semantic representations.

### Comparison among KAGN variants

In order to determine the relative importance and validity of attention mechanism and GCN module of KAGN, we designed the following variants of KAGN and performed a series of ablation studies on different parts of the model. The experimental results are shown in Table 7.

- PTE: Only using posts text encoder (PTE) to extract text features from posts for rumor detection.
- PTE + KAE: Removing knowledge graphs encoder (KGE) of KAGN for rumor detection.
- PTE + KGE: Removing knowledge attention encoder (KAE) of KAGN for rumor detection.
- KAGN: consists of PTE, KAE and KGE for rumor detection.

**Table 7:** Results of comparison among different variants of KAGN on twitter datasets

Dataset	Variant	Accuracy	Precision	Recall	F1
Twitter15	PTE	0.8036	0.8053	0.8036	0.8037
	PTE + KGE	0.8406	0.8486	0.8408	0.8417
	PTE + KAE	0.8636	0.8690	0.8628	0.8642
	KAGN	0.8923	0.8947	0.8905	0.8956
Twitter16	PTE	0.8295	0.8364	0.8296	0.8315
	PTE + KGE	0.8672	0.8734	0.8676	0.8650
	PTE + KAE	0.8750	0.8749	0.8742	0.8744
	KAGN	0.9013	0.9034	0.9062	0.8976
PHEME	PTE	0.8125	0.8056	0.8125	0.8052
	PTE + KGE	0.8281	0.8228	0.8281	0.8208
	PTE + KAE	0.8594	0.8594	0.8594	0.8594
	KAGN	0.8646	0.8402	0.8293	0.8344
Politifact	PTE	0.8203	0.8203	0.8210	0.8202
	PTE + KGE	0.8438	0.8434	0.8434	0.8438
	PTE + KAE	0.8672	0.8683	0.8672	0.8673
	KAGN	0.8790	0.8768	0.8780	0.8751

**Fig. 4:**
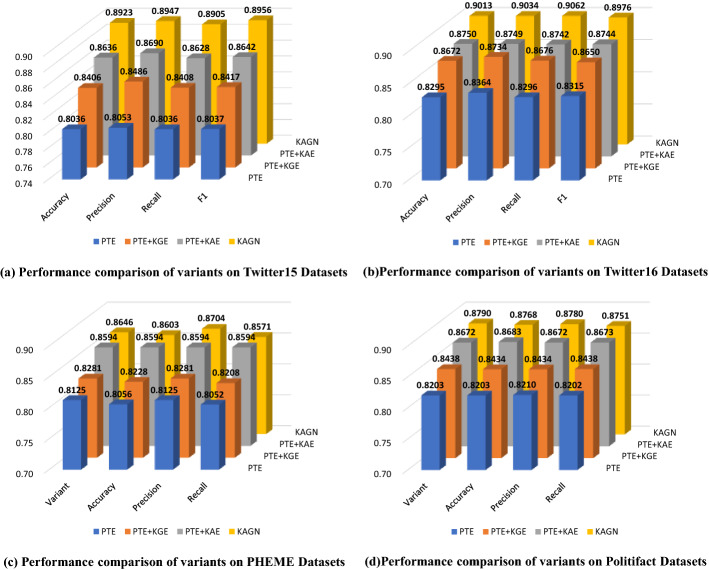
Results of comparison among different variants of KAGN

From the Table 7 and Fig. 4, we can have the following observations: according to the results of four datasets, the variants that are equipped with external knowledge information (KAGN, KGE and KAE) perform significantly better than the non-knowledge variant (PTE). In this case, it appears that entities and concepts extracted from the external knowledge base play an important role in the detection of rumor.

- When we compare the performance of PTE + KAE with that of KAGN, we can see that removing the KGE module reduces the variant's performance by 2.87%, 2.63%, 0.52% and 1.18%, respectively, on the four datasets. Despite the fact that the introduction of KGE does not result in a significant increase in performance, it can provide long-range implicit semantic relationships information between knowledge, which will be beneficial to our model.
- Comparing the performance of the PTE + KGE variant to that of the KAGN variant reveals that removing the KAE module degrades performance across all datasets, with an accuracy loss of 5.17%, 3.41%, 3.65% and 3.52% on four datasets, respectively. We can see that the PTE + KAE model equipped with explicit knowledge information consistently outperforms the PTE + KGE model integrating implicit knowledge, but that both modules provide important complementary information for rumor detection when combined.

In addition, we further investigate the effectiveness and significance of entity and concept knowledge in KAGN. The variants of KAGN are as follows:

- KGE: is a graph convolutional encoder that considers both entity and concept knowledge.
- KGE/E: is a variant of KGE without considering entity knowledge.
- KGE/C: is a variant of KGE without using entity-related concepts knowledge.
- KAE: is an attention encoder that takes account of both entity and concept knowledge.
- KAE/E:is a variant of KAE that not considering entities knowledge.
- KAE/C: is a variant of KAE that eliminates entity-related concepts knowledge.

**Fig. 5:**
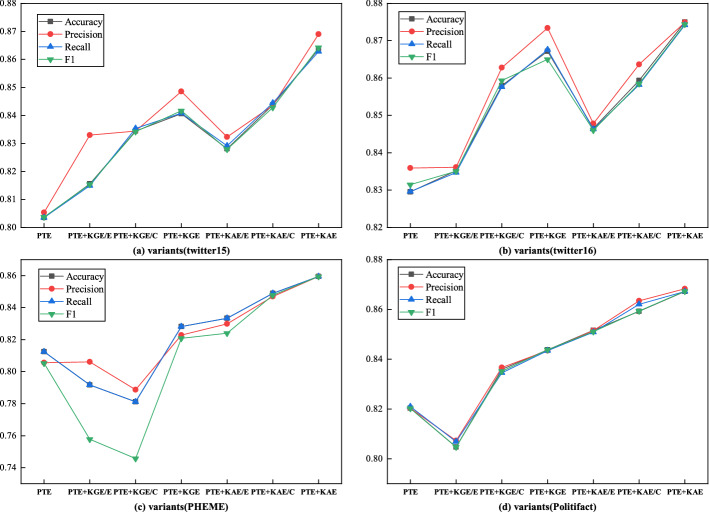
Impact of entity and concept knowledge on model performance for four datasets

Tables 8, 9, 10, 11, and Fig. 5 illustrate the model's effects of entity and concept knowledge, respectively, from which the following conclusions can be drawn:

- When we do not incoporate entity knowledge, the performance of PTE + KGE/E is significantly worse than PTE + KGE, with accuracy decreasing by 2.5%, 3.2%, 4.68% and 3.91% on four datasets, respectively. Similarly, the accuracy of PTE + KAE/E decreases by 3.55%, 2.85%, 2.61% and 1.56% respectively, when compared to PTE + KAE on four dataset. The findings indicate that entity knowledge plays an important role in sentence disambiguation and contributes to a correct understanding of text meaning.
- When concept knowledge is not taken into account, the accuracy of PTE + KGE/C decreases by 0.62%, 0.92%, 3.64% and 1.56% on four datasets respectively compared to PTE + KGE, and the accuracy of PTE + KAE/C decreases by 2%, 1.6%, 1.01% and 0.79% compared to PTE + KAE on four datasets. This suggests that the conceptual knowledge implicit in the text aids comprehension of the text's content.
- Simultaneously, we discovered from the results that entity knowledge has a greater impact on model accuracy. It is possible that entities originate from the text itself, and that entities are more semantically connected to the text, whereas there are numerous entity-based concepts that may cause interference with the text's semantics.

**Table 8:** Impact of entity and concept knowledge on model performance for twitter15 datasets

Variants	Accuracy	Precision	Recall	F1
PTE	0.8036	0.8053	0.8036	0.8037
PTE + KGE/E	0.8156	0.833	0.8149	0.8154
PTE + KGE/C	0.8344	0.8344	0.8354	0.8342
PTE + KGE	0.8406	0.8486	0.8408	0.8417
PTE + KAE/E	0.8281	0.8323	0.8291	0.8279
PTE + KAE/C	0.8438	0.8436	0.8445	0.8428
PTE + KAE	0.8636	0.869	0.8628	0.8642

**Table 9:** Impact of entity and concept knowledge on model performance for twitter16 datasets

Variants	Accuracy	Precision	Recall	F1
PTE	0.8295	0.8359	0.8296	0.8315
PTE + KGE/E	0.8352	0.8361	0.8347	0.8350
PTE + KGE/C	0.8580	0.8628	0.8577	0.8594
PTE + KGE	0.8672	0.8734	0.8676	0.8650
PTE + KAE/E	0.8465	0.8478	0.8463	0.8460
PTE + KAE/C	0.8594	0.8637	0.8582	0.8585
PTE + KAE	0.8750	0.8749	0.8742	0.8744

**Table 10:** Impact of entity and concept knowledge on model performance for PHEME datasets

Variants	Accuracy	Precision	Recall	F1
PTE	0.8125	0.8056	0.8125	0.8052
PTE + KGE/E	0.7813	0.7886	0.7813	0.7457
PTE + KGE/C	0.7917	0.8061	0.7917	0.7578
PTE + KGE	0.8281	0.8228	0.8281	0.8208
PTE + KAE/E	0.8333	0.8298	0.8333	0.8241
PTE + KAE/C	0.8490	0.8469	0.8489	0.8477
PTE + KAE	0.8594	0.8594	0.8594	0.8593

**Table 11:** Impact of entity and concept knowledge on model performance for Politifact datasets

Variants	Accuracy	Precision	Recall	F1
PTE	0.8203	0.8203	0.8210	0.8202
PTE + KGE/E	0.8047	0.8074	0.8068	0.8047
PTE + KGE/C	0.8359	0.8367	0.8345	0.8351
PTE + KGE	0.8438	0.8434	0.8434	0.8438
PTE + KAE/E	0.8516	0.8515	0.8509	0.8511
PTE + KAE/C	0.8593	0.8634	0.8620	0.8593
PTE + KAE	0.8672	0.8683	0.8672	0.8673

The above results show that introducing external knowledge is important to guide rumor detection.

## Complexity analysis

In the knowledge distillation stage, we extracted the set of entities in the text with a confidence level greater than 0.1, as well as the concepts of the top 10 rankings for each entity. Since this is a one-off pre-processing step, it does not need to be repeated in the training loop. The computational complexity and spatial complexity of knowledge extraction is fixed. Table 12 displays the memory usage of the entity and concept sets on four datasets.

**Table 12:** Comparisons of memory usages on four datasets

Memory space	Twitter15	Twitter16	PHEME	Politifact
Entities set	362 KB	199 KB	206 KB	449 KB
Concepts set	1251 KB	313 KB	728 KB	2104 KB

Now, we consider the steps and their time complexity in one iteration. In the Posts Texts Encoder, the vector representations of source post are fed into the multi-filter CNN layers. For a single CNN layer, it costs a computational complexity of $O\left( {knd} \right)$, where $k$ is the kernel size of convolutions, $n$ is the sequence length and $d$ is the representation dimension. The vector representations of entities and concepts are fed into the Knowledge Attention Encoder, and then passed through the bi-LSTM and multi-headed attention mechanisms to obtain the fused representations of knowledge. Bi-LSTM take $O\left( {m^{2} } \right)$ time, where $m$ is the hidden size. Multi-head self/cross attention takes $O\left( {n^{2} d} \right)$ time, where $n$ is the sequence length and $d$ is the representation dimension. In the Knowledge Graph Encoder, the GCN algorithm require a time complexity of $O\left( {l\left\| {A_{0} } \right\|d + lnd^{2} } \right)$ and space complexity $O\left( {lnd + ld^{2} } \right)$, where $l$ is number of layers, $n$ is number of nodes, $\left\| {A_{0} } \right\|$ is number of nonzeros in the adjacency matrix, and $d$ is dimention of features. For memory complexity, $ld^{2}$ is for storing $\left\{ {W^{(l)} } \right\}\left| {_{l = 1}^{L} } \right.$ and the other term is for storing embeddings. For simplicity we omit the memory for storing the graph (GCN) or sub-graphs (other approaches) since they are fixed. The number of nodes of post-entity-concept graph on four datasets are illustrated in Table 13. In addition, we investigate the computational complexity of the model by tracking the training and prediction times of KAGN, which are presented in minute, second, and millisecond (MM:SS.ms) formats as seen in Table 14.

**Table 13:** Number of nodes for post-entity-concept graph on four datasets

Type of nodes	Twitter15	Twitter16	PHEME	Politifact
Post node	1490	818	2018	745
Entities node	1951	1133	1316	3438
Concepts node	4643	1078	4715	12514

**Table 14:** Training and prediction time of KAGN on four datasets

	Twitter15	Twitter16	PHEME	Politifact
training time per epoch (MM:SS.ms)	00:03:233	00:01:120	00:04:360	00:05:580
prediction time per epoch (MM:SS.ms)	00:00:336.7	00:00:167.6	00:00:452.2	00:00:863.9

## Discussion

### Power of knowledge

**Fig. 6:**
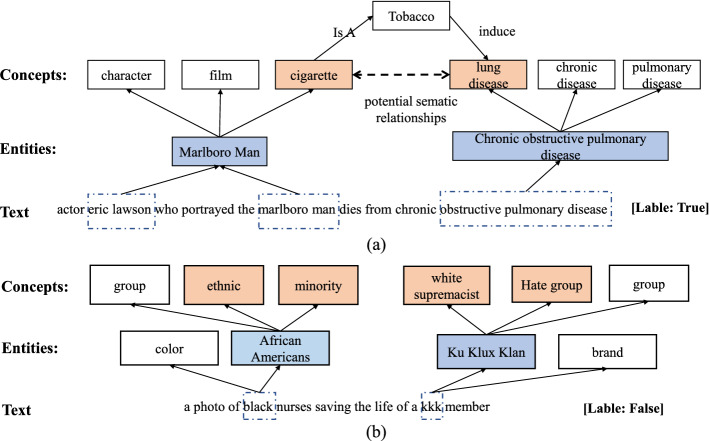
Two examples for power of knowledge

**Fig. 7:**
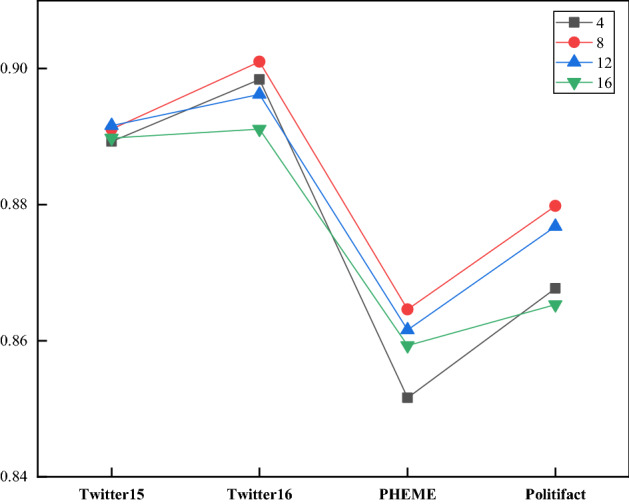
The experimental results of the KAGN under different numbers of cross attention heads

**Fig. 8:**
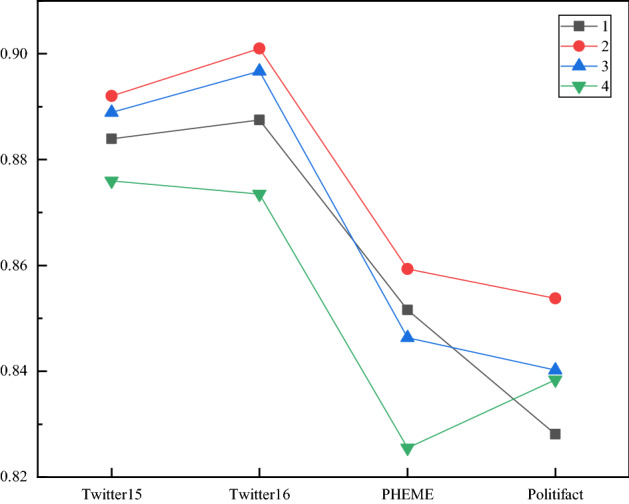
The experimental results of the KAGN under different numbers of GCN layers

We employ entity and conceptual information as prior knowledge to enrich the representation of text posts and enhance classification performance. To verify the efficacy of knowledge in our model, we select some examples from the Twitter dataset for testing and display them in Fig. 6. Our model correctly classifies these texts, whereas traditional DNNs that lack knowledge misclassify them. In general, the information of entities and concepts plays a crucial role in the classification of short texts, especially when context is insufficient. As the first example shown in Fig. 6(a), “eric lawson” stands for person’s name, cannot provide more information, and thus it is challenging to acquire a good representation of A, resulting in the poor performance of conventional DNNs. However, our model is helpful to avoid ambiguity and makes semantics more explicit by integrating entity and concept knowledge. “eric lawson” and “marlboro man” are semantically linked by the identified entity “Marlboro Man”. The concepts such as “character” and “cigarette” further enriches the meaning of the entity. In addition, there may be some underlying relationship between the concepts contained in the sentence, such as “cigarette” and “lung disease”. Our model can mine such long-range semantic associations by building post-entity-concept graphs. Figure 6(b) illustrates another example, “kkk” is a rare word, i.e., occurs less frequently in the training set. However, our model solves the rare and unknown word problem in some degree by introducing knowledge from KB. “kkk” is linked to the entity “Ku Klux Klan”, which conceptually means “white supremacist group” and “hate group”. Furthermore, the MAE module in our model determines that the interpretation of “black” prefers entity “African Americans” based on attention weights. The concepts “ethnic group” and “minority group” further complement “African Americans” semantically. Since “black” and “kkk” are in conflict in terms of high level semantics, which is beneficial for classifying the short text into the correct class (Fig. 7, 8).

### Effects of the numbers of cross attention heads and GCN layers

In this section, we investigate how the numbers of cross attention heads in the Knowledge Attention Encoder and the maximum number of GCN layer affect the model’s performance. Since the number of cross attention heads must be divisible by the word vector dimension, we set the range of the number of heads to [4, 8, 12, 16]. Table 7 shows the performance of the KAGN for different numbers of heads. Although the number of cross attention heads has little effect on the results, we can observe that the performance of the KAGN increases with the number of heads up to 8. We set the range of the number of KAT layers to [1–4]. Table 8 shows the performance of the KAGN for different numbers of GCN layers. We observe that as the number of GCN layers increases, the model performance is not improved or becomes even slightly worse. Hence, we set the numbers of GCN layers and cross attention heads to 2 and 8, respectively.

## Limitation

**Fig. 9:**
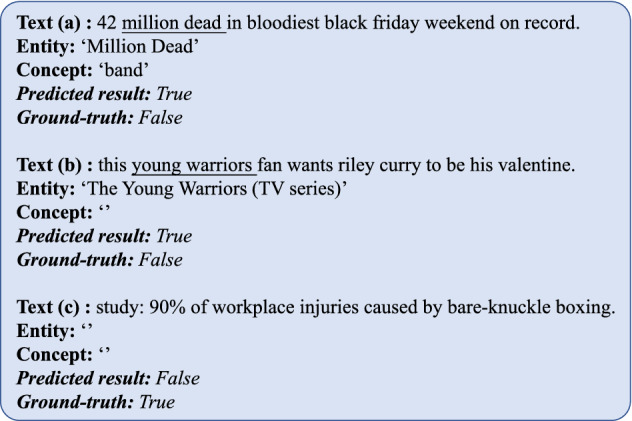
Three failure examples of the proposed KAGN on the Twitter dataset

According to the above experimental results and discussions, our KAGN performs well for rumor detection tasks. However, since the proposed method takes advantage of knowledge from the external knowledge base, one limitation of our method is that the performance of KAGN is influenced by the accuracy of external entity linking tools and knowledge bases, which is beyond our control. Furthermore, KAGN is more applicable to text with obvious entity mention. The ground-truth and predicted results of two samples are shown in Fig. 9. It can be observed from the figure that the entity links in text(a) and text(b) are incorrectntity, and there are no obvious entities in text(c). Therefore, we can only rely on the the word features of texts in our method for classification. However, from the analysis in Table 7 the knowledge of text has an important contribution to the proposed method. Therefore, our method may be limited in predicting the authenticity of the news evoked by texts without obvious entities, which is consistent with the conclusion in Table 7

## Conclusion and future work

KAGN is proposed in this paper as a method to detect rumor, which incorporates entities and concepts from an external knowledge base to complement the semantic representation of the short text of posts. When we incorporate entities and concepts into the representation of the text, we are able to make better use of external knowledge information because we have used an attention mechanism. In addition, we use graph convolutional neural networks to construct graphs containing post texts, entities, and concepts to obtain associative features among knowledge. The experimental results on four publicly datasets demonstrate the effectiveness of the proposed model and that the performance of the model can be effectively improved by introducing external knowledge. In the future, we intend to investigate the combination of multimodal data (e.g.images) and external knowledge for the detection of fake message.

## Data Availability

The datasets used and/or analyzed during the current study are available from the corresponding author on reasonable request.

## Abbreviations

KAGN: Knowledge-powered attention and graph neural networks
CNN: Convolutional neural network
BI-LSTM: Bi-directional long short-term memory
GAN: Generative adversarial network
KGs: Knowledge graphs
GNN: Graph neural networks
GCN: Graph convolutional networks

## Footnotes

1 www.dropbox.com/s/7ewzdrbelpmrnxu/rumdetect2017.zip?dl=0

2 https://www.zubiaga.org/datasets/

3 https://github.com/KaiDMML/FakeNewsNet
